# Longitudinal epigenetic predictors of amygdala:hippocampus volume ratio

**DOI:** 10.1111/jcpp.12740

**Published:** 2017-05-08

**Authors:** Esther Walton, Charlotte A.M. Cecil, Matthew Suderman, Jingyu Liu, Jessica A. Turner, Vince Calhoun, Stefan Ehrlich, Caroline L. Relton, Edward D. Barker

**Affiliations:** ^1^ Department of Psychology Institute of Psychiatry, Psychology & Neuroscience King's College London London UK; ^2^ Department of Psychology Georgia State University Atlanta GA USA; ^3^ Medical Research Council Integrative Epidemiology Unit University of Bristol Bristol UK; ^4^ The Mind Research Network Albuquerque NM USA; ^5^ Department of Electrical Engineering University of New Mexico Albuquerque NM USA; ^6^ Division of Psychological and Social Medicine and Developmental Neurosciences Faculty of Medicine TU Dresden Dresden Germany

**Keywords:** DNA methylation, methylome‐wide, amygdala, hippocampus, longitudinal, Avon Longitudinal Study of Parents and Children

## Abstract

**Background:**

The ratio between amygdala:hippocampal (AH) volume has been associated with multiple psychiatric problems, including anxiety and aggression. Yet, little is known about its biological underpinnings. Here, we used a methylome‐wide approach to test (a) whether DNA methylation in early life (birth, age 7) prospectively associates with total AH volume ratio in early adulthood, and (b) whether significant DNA methylation markers are influenced by prenatal risk factors.

**Methods:**

Analyses were based on a subsample (*n* = 109 males) from the Avon Longitudinal Study of Parents and Children, which included measures of prenatal risk, DNA methylation (Infinium Illumina 450k), T1‐weighted brain scans and psychopathology in early adulthood (age 18–21). Amygdala and hippocampus measures were derived using Freesurfer 5.3.0. Methylation markers related to AH volume ratio across time were identified using longitudinal multilevel modeling.

**Results:**

Amygdala:hippocampal volume ratio correlated positively with age 18 psychosis‐like symptoms (*p *=* *.007). Methylation of a probe in the gene *SP6* associated longitudinally with (a) higher AH volume ratio (FDR *q*‐value = .01) and (b) higher stressful life events during pregnancy (*p *=* *.046). *SP6* is expressed in the hippocampus and amygdala and has been implicated in cognitive decline in Alzheimer's disease. The association between *SP6* DNA methylation, AH volume ratio and psychopathology was replicated in an independent dataset of 101 patients with schizophrenia and 111 healthy controls.

**Conclusions:**

Our findings suggest that epigenetic alterations in genes implicated in neurodevelopment may contribute to a brain‐based biomarker of psychopathology.

## Introduction

Structural alterations in amygdala and hippocampus volume have been linked to a range of psychiatric problems, including anxiety, depression, and aggressive behavior (Campbell, Marriott, Nahmias, & MacQueen, 2004; Pardini, Raine, Erickson, & Loeber, 2014; Schmaal et al., 2016). However, the effect sizes reported so far have been small (Cohen's *d* < 0.2) and findings mixed with regard to the directionality and stability of effects. For example, when comparing patients with depression versus controls, some studies have reported a volume increase (Visser et al., 2014), others a decrease (Matthies et al., 2012; Thijssen et al., 2015) and others still only acute‐disease state associations (Ahdidan et al., 2011; Arnone et al., 2013).

Considering that these structures are both part of the limbic system and are structurally and functionally connected, examining these subcortical structures in the context of one another may be more informative than studying each region in isolation. For instance, a study by Gerritsen et al. (2012) found that a larger amygdala:hippocampal (AH) volume ratio associated with more severe biases toward negative memories (an important cognitive marker of depression) in nondepressed controls, while only weak associations were found when assessing each structure separately. Specifically, compared with persons without negative memory bias, persons with negative memory bias had a 6.3% higher AH volume ratio, but only a 2.2% larger amygdala volume and 3.7% smaller hippocampal volume. Similarly, an increased AH volume ratio (rather than structure‐specific effects) was found to be correlated strongly (*r* = .62) with severity of anxiety in medication‐naive first‐episode patients with depression (MacMillan et al., 2003). Both studies point toward medication‐independent effects, which seem to predate illness onset and might hence be a marker of disease vulnerability. In line with this, a study by Gilliam et al. (2014) reported a weak association (*r* = .19) between AH volume ratio and aggressive behavior (but not depression) in a community sample of young men. The study also found that AH volume ratio mediated the association between maternal depression and later aggression, pointing to a potentially important role of the early environment in AH volume ratio and associated psychopathological outcomes.

Despite these promising findings, little is currently known about the biological underpinnings of an AH volume ratio, as previous models of psychopathology have primarily focused on the biological mechanisms involved in hippocampal atrophy. For instance, the ‘neurotrophic hypothesis of depression’ postulates that chronic hyperactivity of the hypothalamic–pituitary–adrenal axis – often due to environmental stress exposure – leads to increased glucocorticoid levels that can downregulate the activity of growth factors such as BDNF, which over time can result in brain atrophy (Campbell & MacQueen, 2004). Considering that glucocorticoid receptor expression is high in the hippocampus (Sapolsky, Krey, & McEwen, 1984), this region might be particularly damaged. However, evidence suggests that the amygdala may also be affected. For example, Govindarajan et al. (2006) studied transgenic mice, which overexpressed BDNF, and observed (a) increased anxiety‐like behavior and spinogenesis of the amygdala and (b) decreased depressive symptoms and hippocampal atrophy. In line with these antagonistic findings, Manna et al. (2015) reported BDNF levels in healthy elderly individuals to be correlated positively with mean diffusivity in the amygdala, but negatively with that in the hippocampus. These findings suggest that across certain imaging modalities hippocampal deficiency in depression might be inversely linked to amygdala deficits (i.e., a larger AH volume ratio). That is, both structures form an interconnected network, which jointly responds to changes in biological signals.

The mechanisms underlying environmental, stress‐related effects on an AH volume ratio remain unclear. Epigenetic processes, such as DNA methylation, have recently emerged as a candidate mechanism for biological embedding. Epigenetic profiles of the hippocampus and the amygdala have been examined in healthy controls and in relation to a range of psychiatric disorders such as schizophrenia and depression. Taking a gene set enrichment approach using methylome‐wide data, Hass et al. (2015) found altered epigenetic patterns in the *hsa‐miR‐219a‐5p* microRNA target gene set to correlate with hippocampal volume in a schizophrenia sample. Methylation in the serotonin transporter gene *SLC6A4*, a risk locus for depression, has been found to associate with hippocampal grey matter volume and parts of the amygdala in healthy controls (Dannlowski et al., 2014). Similar effects have been observed in patients with depression (Booij et al., 2015), where methylation also related to childhood abuse. With respect to prenatal risk factors, several longitudinal studies have found that exposures such as parental social risks, maternal adiposity, and smoking during pregnancy influence offspring DNA methylation (Cecil et al., 2014; Richmond et al., 2015; Sharp et al., 2015). However, to our knowledge no study so far has examined the prospective epigenetic correlates of an AH volume ratio and how these may connect to the early‐life environment. Moreover, given that DNA methylation is dynamic across the life span, findings of any cross‐sectional study might only apply to the time period under investigation (Martino et al., 2013). It is, therefore, not surprising that in the few existing longitudinal epigenetic studies, most markers identified were not found to consistently associate with prenatal exposures across time points (Cecil et al., 2014; Richmond et al., 2015; Rijlaarsdam et al., 2017; Sharp et al., 2015). Considering that subcortical brain maturation is a continuous process during development (Østby et al., 2009), we were interested in DNA methylation markers that are robustly and longitudinally associated with AH volume ratio. Consequently, in this study we employed a methylome‐wide approach to test (a) whether DNA methylation in childhood (birth and age 7) prospectively associates with total AH volume ratio in early adulthood, and (b) whether significant DNA methylation markers are influenced by prenatal risk factors.

## Methods

### Participants

Participants were drawn from the Accessible Resource for Integrated Epigenomics Studies (ARIES, www.ariesepigenomics.org.uk, Relton et al., 2015), a study nested within the *Avon Longitudinal Study of Parents and Children* (ALSPAC) that contains DNA methylation data for a subset of 1,018 mother–offspring pairs. ALSPAC is an ongoing epidemiological study of children born from 14,541 pregnant women residing in Avon, UK, with an expected delivery date between April 1991 and December 1992 (85% of eligible population; Fraser et al., 2013). Ethical approval for the study was obtained from the ALSPAC Ethics and Law Committee and the Local Research Ethics Committees. The original sample is representative of the general population (Boyd et al., 2013). Please note that the study website contains details of all the data that is available through a fully searchable data dictionary: http://www.bris.ac.uk/alspac/researchers/data-access/data-dictionary/.

#### Subsample with brain magnetic resonance imaging

A total of 507 male participants underwent brain MR imaging between the age of 18–21 years (Mean ± *SD*: 235.5 months ± 10.1 months, range: 216–258 months). Only male participants were scanned owing to the focus of the NIH grant funding this work (R01MH085772‐01A1; Axon, Testosterone and Mental Health during Adolescence). Participants were selected based on their current domicile being within a 3‐hr journey (one‐way) from the scanning site. We excluded 14 participants due to a failure to pass quality control of FreeSurfer‐based image‐analysis pipeline (see below). For this study, we included only youth from ARIES who had available data on brain measures (age 18–21) as well as epigenetic data at birth and at age 7. The final dataset consisted of 109 participants (all males).

### Measures

#### DNA methylation data

Five hundred nanograms of genomic DNA from blood (cord at birth; whole at age 7; ARIES sample) was bisulfite‐converted using the EZ‐DNA methylation kit (Zymo Research, Orange, CA). DNAm was quantified using the Illumina HumanMethylation450 BeadChip (Illumina, San Diego, CA) with arrays scanned using an Illumina iScan (software version 3.3.28). Initial data quality control was conducted using GenomeStudio (version 2011.1, Illumina) to determine the status of staining, extension, hybridization, target removal, bisulfite conversion, specificity, nonpolymorphic, and negative controls. Samples and probes that survived initial data quality control using GenomeStudio (version 2011.1, Illumina) and background detection *p*‐value <.05 were quantile normalized using the *dasen* function within the wateRmelon package (wateRmelon_1.0.3; Pidsley et al., 2013) in R and indexed by beta values (ratio of methylated signal divided by the sum of the methylated and unmethylated signal). Probes were removed if they were cross‐reactive, polymorphic, used for sample identification on the array or had a SNP at the single base extension (*n* = 72,068). We also removed participants with non‐Caucasian or missing ethnicity (based on self‐reports; *n* = 61). As a final step, we regressed out chip and cell type as detailed in Houseman et al. (2012) to remove potentially confounding effects. The resulting dataset consisted of a total of 407,462 probes and 828 samples (cord) and 410,075 probes and 903 samples (age 7), before pruning for brain measures (see below). For more information, see section 1.1 in Supporting information.

#### Brain MRI acquisition and preprocessing

Brain MR images were acquired on a 3T magnet (GE, Chicago, IL) using an 8‐channel receiver‐only head coil (GE). T1‐weighted images were obtained using a 3D fast spoiled gradient‐echo sequence using the following parameters: oblique‐axial orientation (plane passing through the anterior–posterior commissures), 1‐mm isotropic, field of view 256 × 192 × 210 mm, TR = 7.9 ms, TE = 3.0 ms, TI = 450 ms and Flip angle = 20°. Segmentation and surface reconstruction quality were assured by manual inspection of all raw brain MRI volumes, segmented volumes in three planes and pial as well as inflated volumes. Fourteen participants’ brain MRI data failed this quality assurance and were removed from the analysis. Hippocampal, amygdala and intracranial volume measures are a standard output of the FreeSurfer 5.3.0 volumetric segmentation (Fischl et al., 2002). For this study, we derived a measure of total AH volume ratio by dividing total (i.e., left and right) amygdala volume by total hippocampus volume.

#### Prenatal environmental risk

Prenatal environmental risk scores were available for 102 participants and created based on maternal reports on four conceptually distinct but related risk domains: (a) Life events (e.g., death in family, accident, illness), (b) Contextual risks (e.g., poor housing conditions, financial problems), (c) Parental risks (e.g., parental psychopathology, criminal involvement, and substance use), and (d) Interpersonal risks (e.g., intimate partner violence, family conflict). For additional details on these measures and the computation of the risk domain scores see Cecil et al. (2014). Information on maternal tobacco smoking for all 109 participants was obtained by self‐reported questionnaires in early, mid, and late pregnancy. Maternal smoking was categorized on the basis of all three questionnaires into ‘never smoked during pregnancy,’ ‘quit as soon as pregnancy was known,’ and ‘continued smoking during pregnancy’. Birth complications (e.g., abruption, preterm rupture, cervical suture) were recorded at the time of birth and available for all 109 participants. Subcategories were dichotomized to contrast mothers with complications (coded 1) versus those without complications (coded 0) and summed across all three categories to represent a combined birth complication factor. For more information see Barker and Maughan (2009) and Rijlaarsdam et al. (2016).

#### Psychiatric symptoms

Depression scores (for 90 participants) from the Moods and Feelings Questionnaire (Angold, Costello, Pickles, & Winder, 1987) and symptoms of emotional problems, conduct problems and hyperactivity (for 96 participants) – based on the Strength and Difficulty Questionnaire (Goodman, 1997) – were available at age 16 as well as a cumulative measure of psychosis‐like symptoms (PLIKS, for 102 participants) at age 18, which was derived by summing the presence (=1) or absence (=0) of suspected or definite symptoms across eight domains (auditory or visual hallucinations, delusions of being spied on/of reference/of control, thoughts of being broadcast, thought insertion/withdrawal) based on self‐report items.

### Statistical analysis

All analyses were performed in R (version 3.0.1, R Development Core Team 2008) using limma (version 3.18.13) with default parameters.

#### Step 1: How do probes across time (birth and age 7) prospectively associate with AH volume ratio in early adulthood?

To identify temporally predictive markers, we ran a longitudinal (birth and age 7), methylome‐wide, multilevel model (eBayes estimation in limma) on all DNA methylation probes present at both time points (*n* = 407,368), controlling for intracranial volume. Correlations between probes across time were estimated using the duplicateCorrelation function in limma, which fits a mixed linear model by REML individually for each probe and returns a consensus correlation, which is a robust average of the individual correlations between time‐repeated probes blocked by subjects. This was then used as an additional input when fitting multiple linear models. Results were then ranked using an empirical Bayes method to reduce the probe‐wise sample variances toward a common value and to increase the degrees of freedom for the individual variances (Smyth, 2004).

This procedure enabled us to (a) identify probes that are associated with AH volume ratio across time (as opposed to one time point only), (b) model methylation data at both time points concurrently, accounting for correlation patterns between them, and (c) increase power to detect effects. The genomic inflation factor lambda and QQ plots were used to compare the methylome‐wide distribution of *p*‐values with the expected null distribution (Figure S1). Probes passing a false discovery rate (FDR) correction threshold of *q* < .05 were considered significant.

#### Step 2: Do probes that prospectively associate with AH volume ratio over time also associate with prenatal risk factors?

As a second step, we analyzed whether prenatal risk exposure (Life events, Contextual risks, Parental risks, and Interpersonal risks) related to DNA methylation probes that were significantly associated with AH volume ratio (from step 1). We again used a mixed‐model longitudinal design, which allowed us to also investigate (a) whether prenatal risk predicted methylation at one or both time points (i.e., main effect of risk); (b) whether methylation changed significantly over time (i.e., main effect of time), and (c) whether risk exposure interacted with time to predict methylation levels (i.e., risk × time). For significant markers, we also tested for indirect effects between prenatal risk and AH volume ratio via DNA methylation using the R package lavaan (version 0.5‐20; Rosseel, 2012). Last, we examined potentially confounding effects of maternal smoking during pregnancy and birth complications on DNA methylation in significant probes at birth.

#### Relevance to brain tissue

To investigate the relevance of the identified DNA methylation markers (as measured in blood tissue) to temporal lobe brain tissue, we used data from two published studies. First, we looked at CpG‐specific correlation estimates using data from paired blood and temporal lobe biopsy samples from 12 epilepsy patients. Blood and living brain biopsy samples were obtained during neurosurgical treatment (i.e., both tissue samples were taken at the same time). For further information, see Walton et al. (2015). Second, we used data from matched DNA samples isolated from premortem whole blood and postmortem entorhinal cortex brain tissue from 71 individuals (http://epigenetics.iop.kcl.ac.uk/bloodbrain).

Furthermore, to investigate whether genes annotated to our top DNA methylation probes are expressed in hippocampal and amygdala tissue, we accessed data from the Allen Human Brain Atlas (http://www.brain-map.org/).

## Results

### Does AH volume ratio relate to psychiatric symptoms in early adulthood?

Amygdala:hippocampal volume ratio was not associated with measures of depression, emotional problems, conduct problems, and hyperactivity at age 16, but a larger AH volume ratio was related to increased psychosis‐like symptoms at age 18 (spearman's *ρ* = .224, *p *=* *.023, *n* = 102).

#### Which DNA methylation markers at cord and age 7 stably predict AH volume ratio in early adulthood?

**Figure 1:**
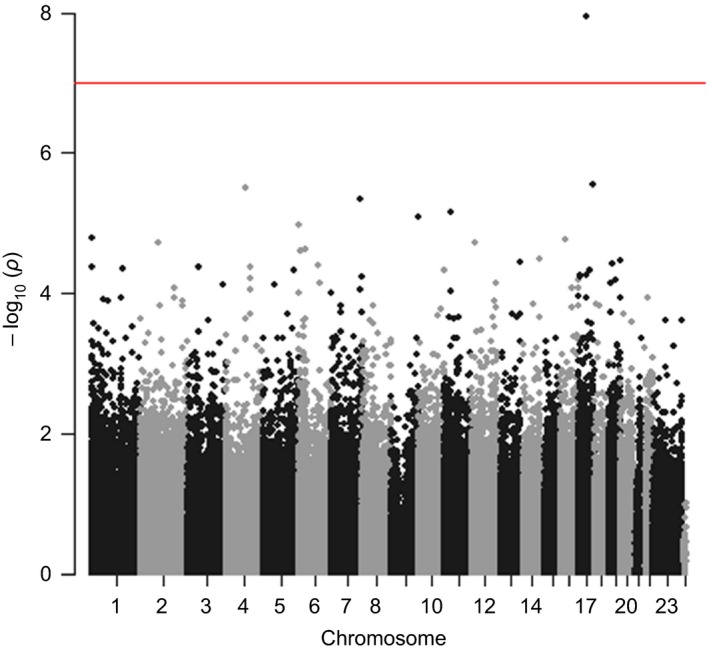
Manhattan plot of methylome‐wide DNA methylation associations (birth and age 7) with amygdala:hippocampus volume ratio (age 18–21), controlling for intracranial volume. Red line indicates methylome‐wide threshold at *p* < 1 × 10^−7^ [Colour figure can be viewed at wileyonlinelibrary.com]

**Figure 2:**
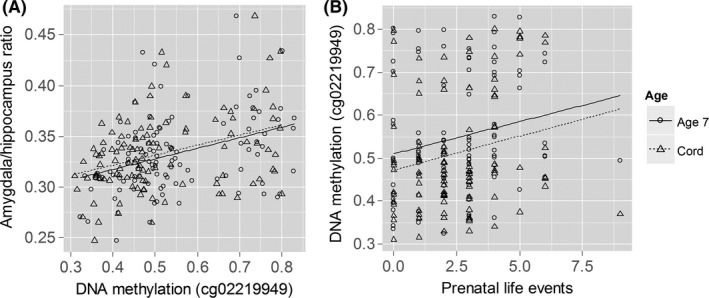
Scatterplots. (A) *SP6*_cg02219949_ DNA methylation both at birth and at age 7 stably predicted amygdala:hippocampus volume ratio at age 18. (B) Prenatal risk life events associated with *SP6*_cg02219949_ DNA methylation both at birth and at age 7. Birth: triangles, dotted regression line; age 7: circles, solid regression line

Inspection of the QQ plot (Figure S1) indicated that test statistics were not overinflated. The most significant differentially methylated probe (DMP), cg02219949, passed both FDR and Bonferroni correction (*p *=* *1.13 × 10^−8^; *q* = .005; *n* = 109; Table 1, Figures 1 and 2A).

**Table 1:** Top 10 differentially methylated probes associated with amygdala:hippocampus volume ratio at birth and age 7

CpG probe	Gene	Chr	Position	Methylation change	*p*‐Value	FDR (*q*)
cg02219949	*SP6*	17	45927392	1.36	1.13 × 10^−8^	.005
cg18384063	*RECQL5*	17	73625493	−0.32	2.83 × 10^−6^	.435
cg18180107	*C4orf37*	4	99064573	0.24	3.21 × 10^−6^	.435
cg08960815	*GIMAP4*	7	150264767	0.88	4.59 × 10^−6^	.467
cg07934552	–	11	32355483	0.30	6.80 × 10^−6^	.548
cg02849279	*CACNA1B*	9	140771661	0.40	8.08 × 10^−6^	.548
cg21242009	–	6	6894182	0.78	1.07 × 10^−5^	.625
cg13614083	*KCNAB2*	1	6085872	−0.27	1.62 × 10^−5^	.723
cg06291594	*SULT1A4*	16	29473557	−0.30	1.71 × 10^−5^	.723
cg25816357	*GOLT1B*	12	21670234	−0.28	1.91 × 10^−5^	.723

Higher methylation of this probe was positively correlated with AH volume ratio across both time points, after controlling for intracranial volume. This finding remained significant in analyses examining each time point separately and was driven by a decrease in hippocampal volume (section 2.2 in Supporting information). At birth, cg02219949 DNA methylation (residualized for chip and cell type) correlated with AH volume ratio (*ρ* = .374, *p *<* *.001) after correction for intracranial volume. This was also the case for cg02219949 DNA methylation at age 7 (*ρ* = .378, *p *<* *.001). Despite the association between AH volume ratio and psychosis‐like symptoms, cg02219949 (at either time point or longitudinally) was unrelated to these symptoms.

Cg02219949 is located in the first intron of Sp6 transcription factor (*SP6)*, a gene implicated in early development, cell proliferation, and previously associated with cognitive decline in Alzheimer Disease (Nakamura et al., 2014; Scohy et al., 2000; Sherva et al., 2014). Regional analysis of all 24 probes annotated to *SP6* revealed six DMPs with nominal effects, three of which passed Bonferroni correction for 24 tests (section 2.3 in Supporting information). These findings further support an association between *SP6* methylation and AH volume ratio. Other DMPs of interest, below multiple comparison corrections, were located in genes such as *KCNAB2* (cg13614083; *p *=* *5.34 × 10^−6^) and *CACNA1B* (cg02849279; *p *= 8.08 × 10^−6^), coding for a voltage‐gated potassium and calcium channel, respectively. Both channels regulate neurotransmitter release and neuronal excitability (Lipscombe, Allen, & Toro, 2013; Schultz, Litt, Smith, Thayer, & McCormack, 1996), and *SULT1A3* (cg06291594; *p *=* *5.10 × 10^−6^), involved in dopamine metabolism and response to glucocorticoids (Bian et al., 2007; Sidharthan, Minchin, & Butcher, 2013). Interestingly, another DMP on chromosome 11 (cg07934552; *p *=* *2.95 × 10^−6^) was found in the proximity of binding sites for several transcription factors, including *NR3C1*, which is also associated with glucocorticoid response (Oberlander et al., 2008).

#### Does the prenatal risk environment predict AH volume ratio‐associated DNA methylation?

We then investigated whether prenatal risks predicted AH volume ratio‐associated *SP6*_cg02219949_ methylation at birth and at age 7. Similar to the previous analysis, we used longitudinal mixed modeling with prenatal risk, time period (cord vs. age 7), and their interaction as predictors for *SP6*_cg02219949_ methylation. Out of the four prenatal risk domains examined (including Life events, Contextual risks, Parental risks, and Interpersonal risks), we found that one risk domain, Life events, was positively associated with *SP6*_cg02219949_ methylation across time at a nominal significance level [*t*(100) = 2.02, *p *=* *.046; *n* = 102; Figure 2B], but the magnitude of correlations at each time point was small (birth: *ρ* = .205, *p *=* *.039; age 7: *ρ* = .202, *p *=* *.042).

While methylation did increase over time [*t*(100) = −2.90, *p *=* *.005], there was no significant time × risk interaction [*t*(100) = 0.19, *p *=* *.849].

As a last step, we tested for a potential indirect effect of *SP6*_cg02219949_ methylation on the association between Life events and AH volume ratio. While Life events were not directly linked to AH volume ratio (*p *=* *.297), there was a trend effect for the indirect path via *SP6*_cg02219949_ methylation at birth and age 7 (standardized path coefficient = .069, *p *=* *.053; *n* = 102; section 2.4 in Supporting information).

Maternal smoking and birth complications might have effects on offspring methylation. Hence, we also investigated potentially confounding effects of smoking and birth complications. *SP6*_cg02219949_ methylation at birth was not associated with maternal smoking during pregnancy [*F*(105) = 2.00, *p *=* *.141, *n* = 109] or birth complications [*F*(105) = 0.55, *p *=* *.652, *n* = 109]. Furthermore, we found no association between gestational age or birth weight and AH volume ratio or *SP6*_cg02219949_ DNA methylation at either time point.

### Replication in a schizophrenia study sample

In light of a link between AH volume ratio and psychosis‐like symptoms, we aimed to replicate the current findings in a cross‐sectional sample of patients with schizophrenia (*n* = 101) versus controls (*n* = 111) featuring measures of DNA methylation in blood and subcortical brain volumes (section 2.5 in Supporting information and Gollub et al., 2013). Because DNA methylation was quantified using the 27k Illumina array, two of the 24 CpGs within the *SP6* gene were available for analysis.

We observed a larger AH volume ratio in patients compared to controls (*b*_stand_ = 0.280, *p *=* *.040, controlling for age, sex, and intracranial volume; See Figure S6), supporting our initial results of an association between AH volume ratio and psychosis‐like symptoms. For one of the two available CpGs – *SP6*_*cg27210136*_ – we observed a small, but significant diagnosis‐by‐CpG interaction effect (*b*_stand_ = 0.318, *p *=* *.020). Specifically, while *SP6*_*cg27210136*_ DNA methylation correlated positively with AH volume ratio in patients, this correlation was negative in healthy controls (see Figure S6).

#### Relevance to brain tissue

To investigate how *SP6*_cg02219949_ DNA methylation in blood correlates to that in brain tissue, we looked up *SP6*_cg02219949_ blood–brain correlation estimates based on two independent resources (see Methods). DNA methylation in *SP6*_cg02219949_ was moderately and significantly (*ρ* = .62, *p *=* *.035) correlated between both tissue types using data from paired blood and temporal lobe biopsy samples. This was confirmed using a second dataset from matched DNA samples isolated from premortem whole blood and postmortem entorhinal cortex brain tissue (*ρ* = .88, *p *<* *.001).

We then investigated whether *SP6* is expressed in hippocampal and amygdala brain tissue using data from the Allen Human Brain Atlas. *SP6* was expressed throughout the hippocampal formation and the amygdala with highest expression values in the CA3 and CA4 region of the hippocampus and the basolateral and ‐medial nucleus of the amygdala (Figure S7).

## Discussion

### Summary

In this study, we used a methylome‐wide approach to test (a) whether DNA methylation in childhood (birth and age 7) prospectively associates with total AH volume ratio in early adulthood, and (b) whether significant DNA methylation markers are influenced by prenatal risk factors. While AH volume ratio related to psychosis‐like symptoms in early adulthood, we found that at birth and age 7, methylation of a probe in the first intron of the gene *SP6* predicted increased AH volume ratio after FDR correction. This probe was nominally associated with increased stressful life events during pregnancy and showed significant convergence with brain tissue methylation.

### DNA methylation and AH volume ratio

*SP6* belongs to a family of transcription factors that bind to DNA motifs often found in promoter, enhancing, and other control regions of many mammalian genes (Scohy et al., 2000). *SP6* is expressed in the developing ectoderm, which differentiates later to form the nervous system as well as tooth enamel and epidermal tissue (Nakamura et al., 2004). Animal studies have found that 20% of *SP6* deficient mice die within 2 month of age and those that survive are smaller than the wildtype and also show basic abnormalities in limb, skin, and tooth development (Nakamura et al., 2008, 2014; Talamillo et al., 2010). Interestingly, a polymorphism in *SP6*, located in the same intron and 3,000 base pairs upstream of the methylation probe identified in this study, was associated with cognitive decline in an Alzheimer disease cohort (*p *=* *7.99 × 10^−8^; Sherva et al., 2014). These studies support our finding of a potentially important role of *SP6* in early neurodevelopment. In our study, *SP6*_cg02219949_ (at either time point or longitudinally) was unrelated to psychosis‐like symptoms, despite an association of AH volume ratio with these symptoms. This may indicate subthreshold effects due to the population design of our study and young age of the participants. Also somewhat surprisingly, we found that AH volume ratio did not – as hypothesized – relate to measures of depression or mood disturbances, but instead to psychosis‐like symptoms. To our knowledge, no study so far has investigated the link between AH volume ratio and psychosis, although amygdala and hippocampal volume reductions are well‐replicated findings in schizophrenia (van Erp et al., 2015). Using a cross‐sectional replication sample of patients with schizophrenia and healthy controls, we were able to further support the link between *SP6* DNA methylation, AH volume ratio, and psychosis. We believe that these results further underline the advantage of using a brain‐based measure (especially in subclinical populations), which might lie closer to the underlying biology than behavioral correlates (Gottesman & Gould, 2003). Furthermore, the fact that methylation levels were found to be significantly correlated between blood and brain tissue across two independent resources supports the relevance of the identified marker to the brain.

None of the other findings survived corrections for multiple comparisons. However, some of the highest ranking probes were associated with genes that may be interesting candidates for follow‐up studies. *KCNAB2* codes for a voltage‐gated potassium channel regulating neurotransmitter release and neuronal excitability. The protein is highly expressed in the hippocampus (Proepper, Putz, Russell, Boeckers, & Liebau, 2014) and deletion of the gene in mice leads to aberrant excitability of neurons in the amygdala and impairments in associative fear conditioning (Perkowski & Murphy, 2011), a trait also observed in depressed patients and to be predictive of later aggressive behavior in children (Gao, Raine, Venables, Dawson, & Mednick, 2010; Otto et al., 2014).

*SULT1A3* is also highly expressed in temporal structures such as the hippocampus (Salman, Kadlubar, & Falany, 2009). This protein is involved in dopamine metabolism (Sidharthan et al., 2013) and has been shown to respond to glucocorticoids (Bian et al., 2007). Glucocorticoids play a key role in risk for psychological syndromes such as depression, anxiety, and antisocial behavior (Haller, 2014) and are also strongly responsive to environmental factors such as stress (McEwen et al., 2015). For instance, a study by Radtke et al. (2015) reported that DNA methylation in the glucocorticoid receptor gene *NR3C1* not only associated with depressive or borderline personality disorder symptoms but was also moderated by childhood maltreatment. Our findings that *SUJLT1A3* and another independent probe, located in the proximity of binding sites for transcription factors including *NR3C1*, showed a suggestive association with AH volume ratio provides preliminary evidence for an involvement of glucocorticoid signaling in brain‐based phenotypes of psychopathology.

### DNA methylation and prenatal environmental risk

DNA methylation in *SP6* was nominally associated with one of the four prenatal risk domains examined – stressful life events during pregnancy. Although results have to be considered with caution and warrant further validation, it is possible that they may be due to the fact that life events were more commonly reported (i.e., higher incidence rates) than other risk domains. Considering that we used an epidemiological population sample, rates for items such as parental psychopathology, partner violence, or substance use were much lower compared to stressful prenatal Life risks (such as death in the family, accidents, or illness). However, it is also possible that there are indeed distinct effects of specific risk domains, which then impact DNA methylation in genes related to early development. A study by van der Waerden, Galéra, Saurel‐Cubizolles, Sutter‐Dallay, and Melchior (2015) – investigating the associations between environmental risk factors and maternal depressive symptom trajectories – reported that not all prenatal risk factors seem to impact depression trajectories to the same degree. That is, some variables were more uniquely associated with a particular trajectory class than others. Whether indeed prenatal life events exert distinct effects on brain‐related DNA methylation patterns will need to be investigated further in future studies.

### Limitations

Results should be interpreted in light of the following limitations. First, the time frame investigated in this study is large, spanning pregnancy to early adulthood. Although we found that DNA methylation in a probe annotated to *SP6* both at birth and at age 7 stably predicted AH volume ratio during early adulthood, future studies are needed to research the precise molecular mechanisms in a time‐sensitive manner. Secondly, the observed effects of methylation on AH volume ratio were rather small, suggesting that there are likely to be other influencing factors (such as genetic effects) in AH volume ratio development. Third, DNA methylation was measured in blood. Although we could provide some evidence that DNA methylation in *SP6*_cg02219949_ is correlated between blood and brain tissue and that *SP6* is expressed in brain tissue, the functional relevance especially with respect to gene expression remains to be investigated in detail. Fourth, our analyses were based on a homogenous sample of adolescent, Caucasian males, and future studies should investigate how well our findings translate to different populations, including females.

In summary, our findings suggest that environmental risk exposure could lead to long‐lasting epigenetic alterations in genes important for neurodevelopment that in turn may contribute to a brain‐based biomarker of psychopathology. These results could help to identify intermediate biomarkers and potential targets for intervention.

**Key points**

- We employed a prospective, epigenome‐wide approach to study epigenetic correlates of a larger amygdala:hippocampal (AH) volume ratio, which was linked to psychosis‐like symptoms.
- The association between DNA methylation and AH volume ratio was replicated in a sample of patients with schizophrenia and healthy controls.
- The study is highly novel in the use of (a) a longitudinal design where DNA methylation precedes brain imaging measures, and (b) the examination of prenatal risk factors effects on DNA methylation.
- Our results point to genes related to neurodevelopmental functions, which may highlight the potential use of epigenetics as biomarkers of brain‐based correlates of psychopathology.

## Supporting information


**Table S1.** Regional results.
**Figure S1.** QQ plot.
**Figure S2.** Correlation of cg02219949 DNA methylation at birth and at age 7.
**Figure S3.** Correlation of cg02219949 DNA methylation at birth with total A) hippocampal and B) amygdala volume.
**Figure S4.** Graphical representation of regional results.
**Figure S5.** Prospective inter‐relationship between prenatal life events, SP6cg02219949 DNA methylation and AH volume ratio.
**Figure S6.** Replication of findings in a cross‐sectional sample of patients with schizophrenia and controls.
**Figure S7.** *SP6* expression values in the human hippocampus and the amygdala.
