# Supplementary material for: Longitudinal epigenetic predictors of amygdala:hippocampus volume ratio
Source: J Child Psychol Psychiatry. 2017 May 8;58(12):1341–50. doi: 10.1111/jcpp.12740 (PMC5677591; doi:10.1111/jcpp.12740)
Supplement: Supplementary file 1 — Table S1. Regional results. Figure S1. QQ plot. Figure S2. Correlation of cg02219949 DNA methylation at birth and at age 7. Figure S3. Correlation of cg02219949 DNA methylation at birth with total A) hippocampal and B) amygdala volume. Figure S4. Graphical representation of regional results. Figure S5. Prospective inter‐relationship between prenatal life events, SP6cg02219949 DNA methylation and AH volume ratio. Figure S6. Replication of findings in a cross‐sectional sample of patients with schizophrenia and controls. Figure S7. SP6 expression values in the human hippocampus and the amygdala. [file JCPP-58-1341-s001.docx]

Online Supplementary Material for - Longitudinal epigenetic predictors of amygdala:hippocampus volume ratio - by Walton et al.

**Methods**

- 1. DNA methylation preprocessing

500ng genomic DNA from blood (cord at birth; whole at age 7) was bisulfite-converted using the EZ-DNA methylation kit (Zymo Research, Orange, CA, USA). DNAm was quantified using the Illumina HumanMethylation450 BeadChip (HM450k; Illumina, USA) with arrays scanned using an Illumina iScan (software version 3.3.28). The protocol followed manufacturer instructions using the recommended alternative incubation conditions for use with Illumina Infinium arrays. Illumina HumanMethylation450 BeadChips (Illumina, San Diego, USA) were run following the manufacturer’s protocol with no modifications. Initial quality control of data generated was conducted using GenomeStudio (version 2011.1) to determine the status of staining, extension, hybridization, target removal, bisulfite conversion, specificity, non-polymorphic and negative controls.

Samples were distributed across slides in a semi-random approach to minimise the potential relationship between batch effects and other variables. During the data generation process a wide range of batch variables were recorded in a purpose-built laboratory information management system (LIMS). The LIMS also reported QC metrics from the standard control probes on the 450k BeadChip for each sample back to the laboratory.

Samples (n_birth_ = 25; n_age 7_ = 8) or probes (n_birth_ = 7873; n_age 7_ = 4861) that failed quality control (>1% probes/samples with background detection p-value >= 0.05) were excluded from further analysis. Sex checks were performed using X/Y chromosome methylation. Genotype probes on the HM450k were compared between samples from the same individual and against SNP-chip data to identify and remove any sample mismatches. Samples were quantile normalised using the *dasen* function within the wateRmelon package (version 1.4.0) in R. **Normalization performance was evaluated using all three testing metrics in wateRmelon (genki assessing SNP-related probes, dmrse assessing imprinted probes and seabi, assessing gender differences).** Probes known to be cross-reactive, polymorphic and SNP probes were removed (n = 72,068). We also removed participants with non-caucasian or missing ethnicity (based on self-reports; n = 61). Cell type proportions (CD8 T lymphocytes, CD4 T lymphocytes, natural killer cells, B lymphocytes, monocytes and granulocytes) for each participant were estimated using the reference-based approach detailed in Houseman et al. (2012).

**Results**

2.1 QQ plot


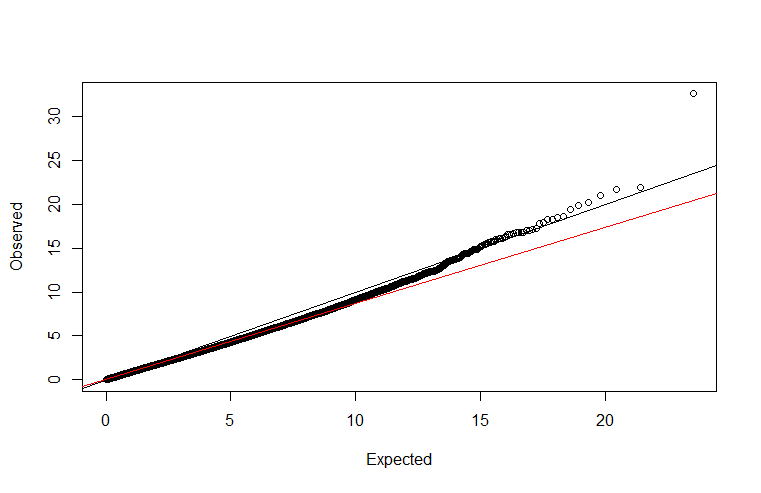


SM Figure 1. QQ plot used to compare the epigenome-wide distribution of P-values with the expected null distribution. The red line shows the fitted slope to the data. The black line represents the theoretical slope without any stratification. A lambda estimate of 0.869 provided no evidence for inflation of test statistic.

2.2 Volume-specific results

Cg02219949 DNA methylation at birth correlated with that at age 7 (rho = .854, p < .001; SM Figure 2).


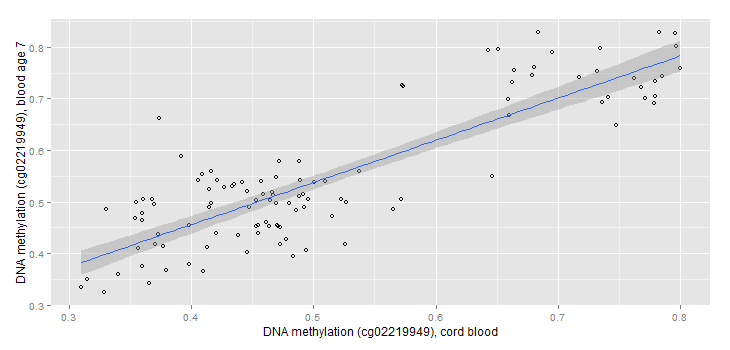


SM Figure 2. Correlation of cg02219949 DNA methylation at birth and at age 7.

Higher methylation of cg02219949 was negatively associated with total hippocampal volume ratio across both time points after controlling for intracranial volume (t = -4.37, *p* = 1.92*10^-5^, SM Figure 3A), with little effect on total amygdala volume (t = 0.75, *p* = 0.45, SM Figure 3B).


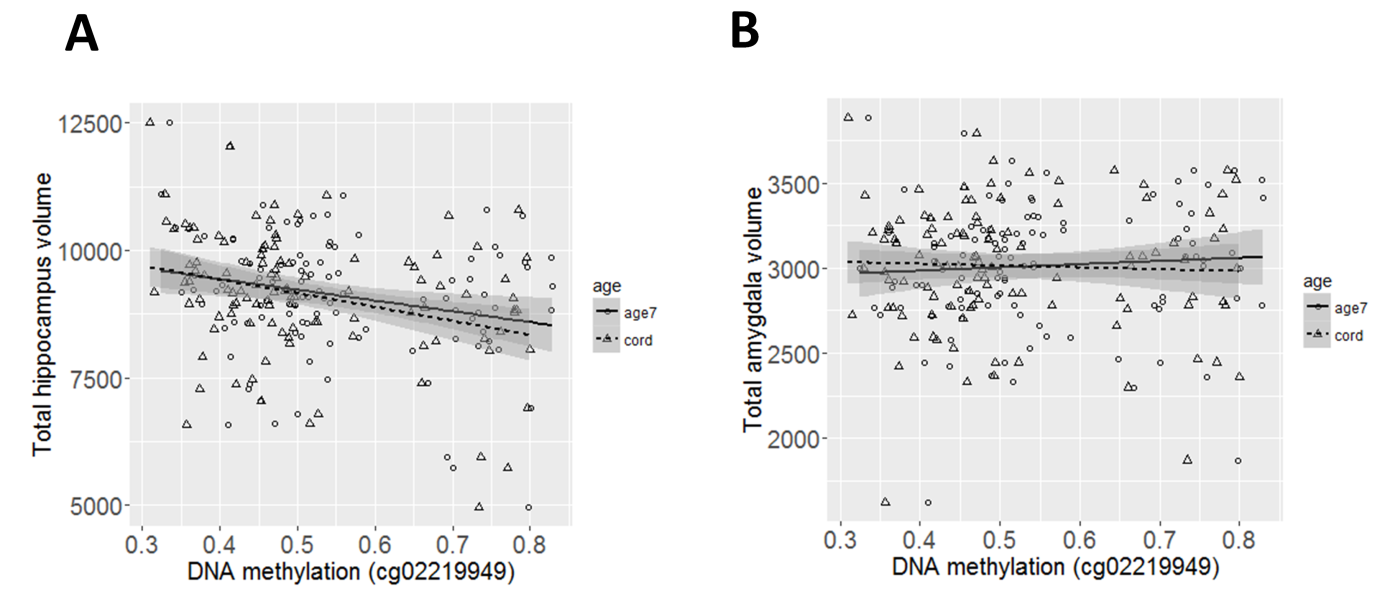


SM Figure 3. Correlation of cg02219949 DNA methylation at birth with total A) hippocampal and B) amygdala volume.

2.3 Regional analysis

We also investigated regional results, including all CpGs within the gene *SP6* (n=24). Six CpGs shows nominal effects and three of these passed Bonferroni correction for 24 test (SM Table 1 and SM Figure 4).

| **CpG probe** | **methylation change** | **P-value** |
| --- | --- | --- |
| cg02219949* | 1.36 | 1.13E-08 |
| cg03029292* | -0.27 | 1.44E-04 |
| cg23391107* | 0.29 | 1.23E-03 |
| cg00598429 | -0.12 | 3.64E-02 |
| cg17842670 | -0.15 | 3.73E-02 |
| cg16779251 | -0.17 | 4.47E-02 |
| cg15951870 | 0.16 | 6.57E-02 |
| cg03668475 | -0.05 | 8.92E-02 |
| cg17733447 | 0.12 | 1.12E-01 |
| cg06138226 | -0.09 | 1.33E-01 |
| cg13230424 | 0.18 | 1.53E-01 |
| cg10531725 | 0.15 | 1.56E-01 |
| cg13457515 | 0.06 | 3.57E-01 |
| cg26888147 | -0.05 | 5.05E-01 |
| cg05044743 | 0.02 | 5.20E-01 |
| cg03071876 | 0.05 | 5.83E-01 |
| cg20020844 | 0.06 | 6.05E-01 |
| cg09439817 | -0.01 | 6.68E-01 |
| cg09965384 | 0.01 | 7.42E-01 |
| cg27210136 | -0.03 | 7.45E-01 |
| cg08974244 | -0.01 | 8.41E-01 |
| cg04663203 | 0.00 | 9.17E-01 |
| cg23355492 | 0.01 | 9.37E-01 |
| cg20556951 | 0.01 | 9.38E-01 |

SM Table 1. Regional results, including all 24 probes annotated to SP6. Bold indicates nominally significant results; * indicate significant results after Bonferroni-correction for 24 test. Methylation change represents effect of unstandardized methylation residuals, corrected for chip and batch effects, on AH volume ratio.


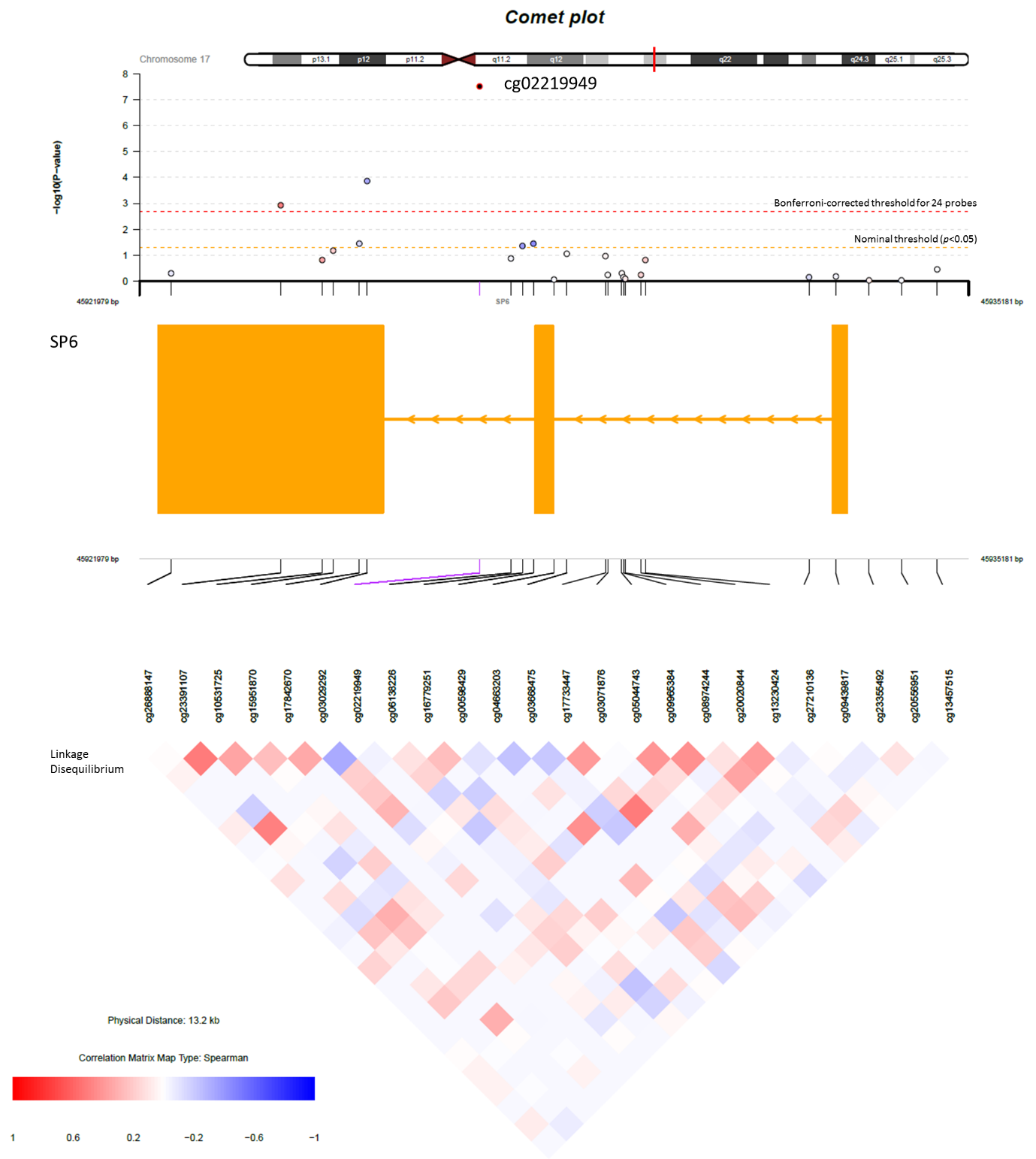


SM Figure 4. Graphical representation of regional results. From top to bottom: Location of analysed region on chromosome 17; Manhattan plot of all 24 probes annotated to *SP6* with –log(p-value) plotted on the y-axis. Two significance thresholds (i.e. the orange and yellow dotted lines) were added for reference; *SP6* gene structure with one intron (line) and one exon (wide box), please note two alternative transcription start sites (thin box); genomic location of all 24 CpGs and correlation plot.

2.4 Mediation analysis


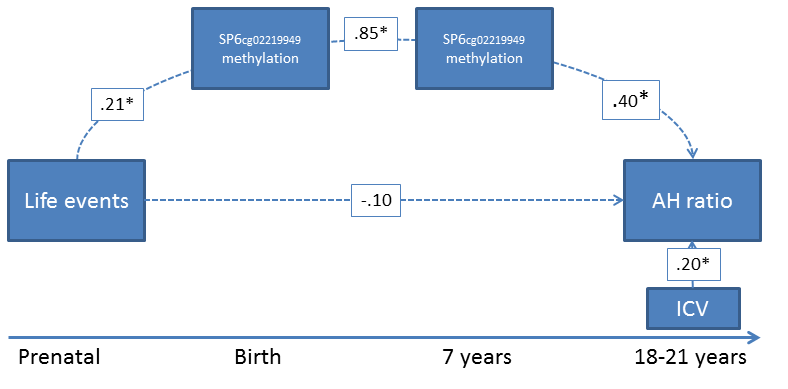


SM Figure 5. Prospective inter-relationship between prenatal life events, *SP6*_cg02219949_ DNA methylation and AH volume ratio. Solid arrowed lines = significant paths; dashed arrowed lines = insignificant paths; * = significant standardized path coefficients; AH ratio = amygdala-hippocampus volume ration; ICV = intracranial volume.

2.5 Replication in a schizophrenia study sample

*Sample description*

Cryo-conserved blood samples from participants of the Mind Clinical Imaging Consortium (MCIC) study of schizophrenia from four participating sites (the University of New Mexico (UNM), the University of Minnesota (UM), Massachusetts General Hospital (MGH), and the University of Iowa (UI)) were used to determine DNA methylation. Patients had a Diagnostic and Statistical Manual of Mental Disorders (*DSM-IV)* diagnosis of schizophrenia, schizophreniform disorder (n=4) or schizoaffective disorder (n=3), established using a Structured Clinical Interview for DSM disorders (SCID; (First, Spitzer, Gibbon, & Williams, 2002)) and a review of case files by trained clinicians. In the initial cohort, controls were matched to the patient group for age, gender, and parental education. Healthy controls were excluded if they had a history of a medical or Axis I psychiatric diagnosis. All subjects gave written informed consent prior to study enrolment. The human subjects research committees at each of the four sites approved the study protocol. For additional details about the participants and clinical measures, see Ehrlich et al. (2010).

*DNA methylation preprocessing*

**Blood samples were obtained from 234 participants and sent to the Harvard Partners Center for Genetics and Genomics for DNA extraction. All DNA extraction, bisulphite modification and hybridization steps were done blinded to group assignment, the latter two were performed at the Mind Research Network Neurogenetics Core Lab on an Infinium HumanMethylation27 BeadChip using Illumina Infinium Methylation Assay. Built-in controls were used to evaluate the quality of individual arrays. CpG sites with a bead count of less than three in five percent of samples or with a detection p-value > 0.05 in at least five percent of samples (n = 70) were removed as well as samples having a detection p-value > 0.05 in at least one percent of CpG sites (n = 0). Subsequently, we performed a quantile color bias adjustment of the red and green fluorescence channel intensities. After removing one intensity outlier, intensities were normalized using watermelon** (Pidsley et al., 2013)**. Next, we removed five samples displaying gender mismatch and corrected our data statistically for the effects of chip and bisulphite conversion efficiency.** After basic quality control procedures, probe intensities were normalized using nanet (instead of danet, because the **Infinium HumanMethylation27 BeadChip** does only have type I beads). Nanet quantile normalizes methylated and unmethylated intensities together, and then calculates betas. This should equalize dye bias. **We also corrected our data statistically for the effects of chip and bisulphite conversion efficiency by first estimating coefficients in** a linear regression model including bisulphite conversion efficiency, chip ID, gender, age and diagnostic group as independent variables, **and then computing corrected beta values regressing out bisulphite conversion efficiency and chip effects only. As a result of this, some residualized beta values were outside a range of 0 to 1. We corrected for this by first adding the lowest value as a constant to all data points, and then dividing by the largest value to ensure methylation values within the range of 0 to 1. The subsequent methylation data set comprised 228 subjects (110 schizophrenia patients and 118 healthy controls) and 27,480 CpG sites. Two CpGs within the *SP6* gene - cg23355492 and cg27210136 - were available for analysis.**

***Brain MRI acquisition and preprocessing***

The T1-weighted structural brain scans at each of the four sites were acquired with a coronal gradient echo sequence: repetition time (TR)=2530 ms for 3T, 12 ms for 1.5T; echo time (TE)=3.79 ms for 3T, 4.76 ms for 1.5T; inversion time (TI)=1100 for 3T; bandwidth=181 for 3T, 110 for 1.5T; 0.625×0.625 voxel size ; slice thickness 1.5 mm; 256×256×128 cm matrix; field of view (FOV) = 16 cm; number of excitations (NEX) = 1 for 3T, 3 for 1.5T.

Data from three consecutive volumes were registered, motion corrected, averaged and analyzed in an automated manner with atlas-based FreeSurfer software suite (http://surfer.nmr.mgh.harvard.edu, Version 4.0.1). This process included volumetric segmentation (Desikan et al., 2006; B Fischl & Dale, 2000; B Fischl, Sereno, & Dale, 1999; Bruce Fischl et al., 2002) and the estimation of total intracranial volume (ICV) (Buckner et al., 2004). Segmentation reconstruction quality were assured by manual inspection of all raw MRI volumes and segmented volumes in three planes. **The final sample with complete structural MRI and DNA methylation data comprised 212 subjects (101 schizophrenia patients and 111 healthy controls).**

***Replication analysis***

**Replication results indicated that** AH volume ratio was larger in patients with schizophrenia (SM Figure 6A) and differentially related to cg27210136 DNA methylation – linked to the gene *SP6* - in patients compared to controls (SM Figure 6B).

**
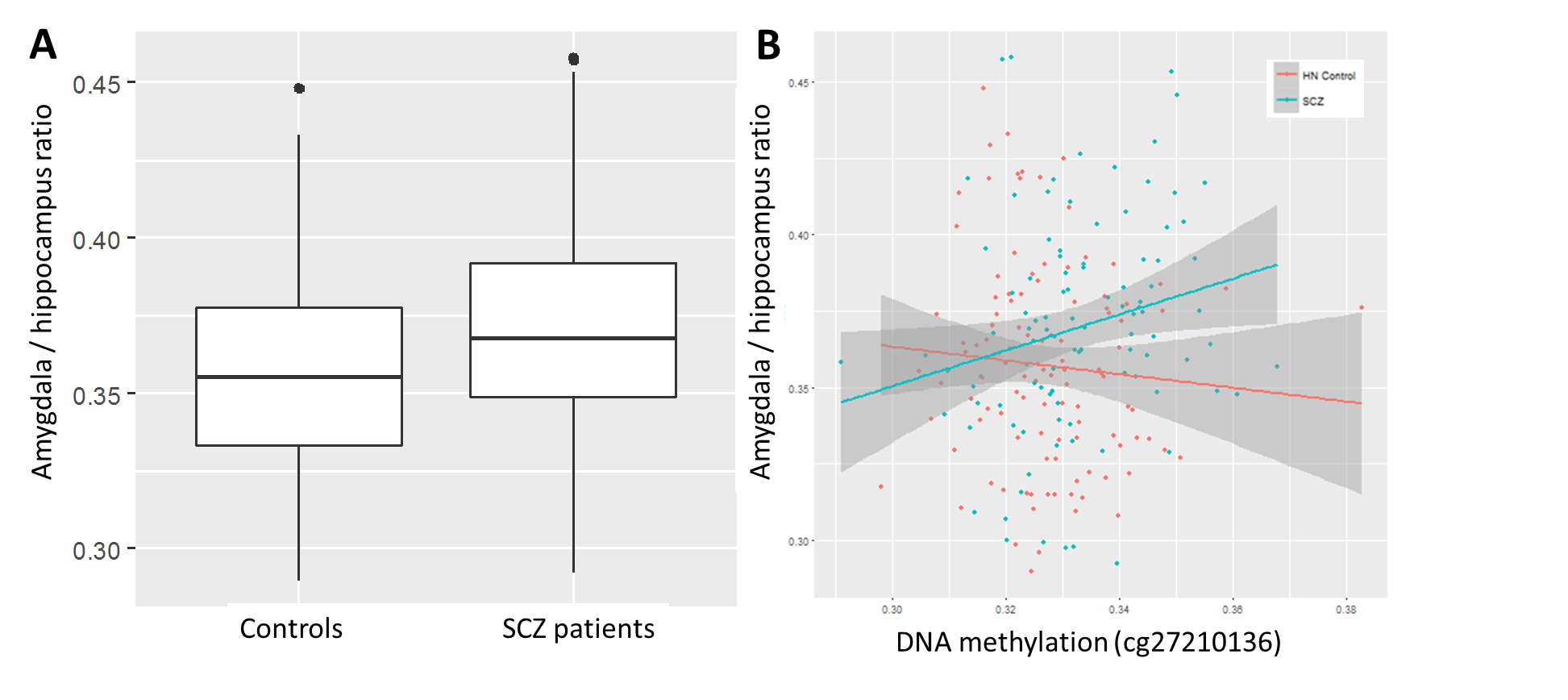
**

SM Figure 6. Replication of findings in a cross-sectional sample of patients with schizophrenia and controls.

2.6 *SP6* expression in human hippocampal and amygdala tissue

To investigate whether *SP6* is expressed in human hippocampal and amygdala tissue, we used publicly available data based on six human brains from the Allen Human Brain Atlas (http://www.brain-map.org and (Hawrylycz et al., 2012)). In detail, we extracted z-scored expression values for two *SP6* probes (CUST_487_PI416573500 and CUST_497_PI416379584) for six hippocampal (dentate gyrus, CA1/CA2/CA3/CA4 fields and subiculum) and six amygdala (amygdalo-hippocampal transition zone, basolateral nucleus, basomedial nucleus, central nucleus, cortico-medial group, lateral nucleus) regions. Z-scores larger than zero (in red) represent higher expression values relative to other brain regions. SM Figure 5 is an exemplary representation of *SP6* expression values in the hippocampus and the amygdala for one of the two *SP6* probes and for one subject.


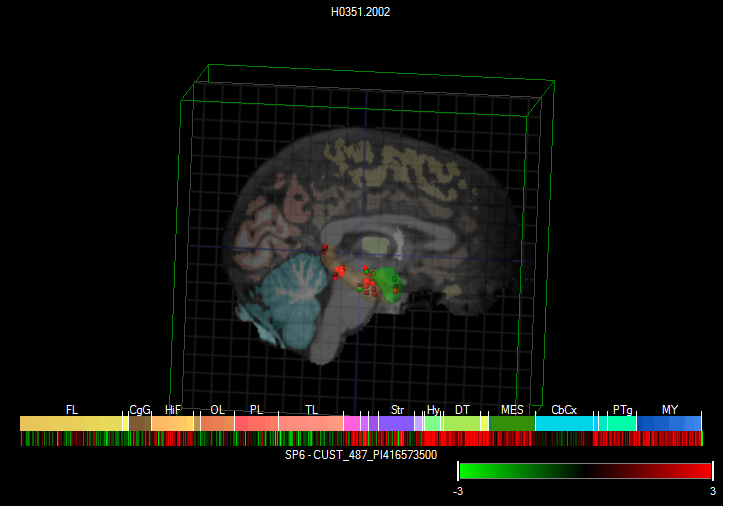


SM Figure 7: *SP6* expression values in the human hippocampus and the amygdala based on data from the Allen Human Brain Atlas.

**References**

Buckner, R. L., Head, D., Parker, J., Fotenos, A. F., Marcus, D., Morris, J. C., & Snyder, A. Z. (2004). A unified approach for morphometric and functional data analysis in young, old, and demented adults using automated atlas-based head size normalization: reliability and validation against manual measurement of total intracranial volume. *NeuroImage*, *23*(2), 724–738. https://doi.org/10.1016/j.neuroimage.2004.06.018

Desikan, R. S., Ségonne, F., Fischl, B., Quinn, B. T., Dickerson, B. C., Blacker, D., … Killiany, R. J. (2006). An automated labeling system for subdividing the human cerebral cortex on MRI scans into gyral based regions of interest. *NeuroImage*, *31*(3), 968–980. https://doi.org/10.1016/j.neuroimage.2006.01.021

Ehrlich, S., Morrow, E. M., Roffman, J. L., Wallace, S. R., Naylor, M., Bockholt, H. J., … Holt, D. J. (2010). The COMT Val108/158Met polymorphism and medial temporal lobe volumetry in patients with schizophrenia and healthy adults. *NeuroImage*, *53*(3), 992–1000. https://doi.org/10.1016/j.neuroimage.2009.12.046

First, M., Spitzer, A., Gibbon, M., & Williams, J. (2002). *Structured Clinical Interview for DSM-IV-TR Axis I Disorders, Research Version, Nonpatient Edition*. New York: New York State Psychiatric Institute.

Fischl, B., & Dale, A. M. (2000). Measuring the thickness of the human cerebral cortex from magnetic resonance images. *Proceedings of the National Academy of Sciences of the United States of America*, *97*(20), 11050–11055. https://doi.org/10.1073/pnas.200033797

Fischl, B., Salat, D. H., Busa, E., Albert, M., Dieterich, M., Haselgrove, C., … Dale, A. M. (2002). Whole brain segmentation: automated labeling of neuroanatomical structures in the human brain. *Neuron*, *33*(3), 341–355.

Fischl, B., Sereno, M. I., & Dale, A. M. (1999). Cortical surface-based analysis. II: Inflation, flattening, and a surface-based coordinate system. *NeuroImage*, *9*(2), 195–207. https://doi.org/10.1006/nimg.1998.0396

Hawrylycz, M. J., Lein, E. S., Guillozet-Bongaarts, A. L., Shen, E. H., Ng, L., Miller, J. A., … Jones, A. R. (2012). An anatomically comprehensive atlas of the adult human brain transcriptome. *Nature*, *489*(7416), 391–399. https://doi.org/10.1038/nature11405

Houseman, E. A., Accomando, W. P., Koestler, D. C., Christensen, B. C., Marsit, C. J., Nelson, H. H., … Kelsey, K. T. (2012). DNA methylation arrays as surrogate measures of cell mixture distribution. *BMC Bioinformatics*, *13*(1), 86. https://doi.org/10.1186/1471-2105-13-86

Pidsley, R., Y Wong, C. C., Volta, M., Lunnon, K., Mill, J., & Schalkwyk, L. C. (2013). A data-driven approach to preprocessing Illumina 450K methylation array data. *BMC Genomics*, *14*, 293. https://doi.org/10.1186/1471-2164-14-293
